# The Proteasome Is a Molecular Target of Environmental Toxic Organotins

**DOI:** 10.1289/ehp.11865

**Published:** 2008-10-23

**Authors:** Guoqing Shi, Di Chen, Guangshu Zhai, Marina S. Chen, Qiuzhi Cindy Cui, Qunfang Zhou, Bin He, Q. Ping Dou, Guibin Jiang

**Affiliations:** 1 State Key Laboratory of Environmental Chemistry and Ecotoxicology, Research Center for Eco-Environmental Sciences, Chinese Academy of Sciences, Beijing, People’s Republic of China; 2 Prevention Program, Barbara Ann Karmanos Cancer Institute, and Department of Pathology, School of Medicine, Wayne State University, Detroit, Michigan, USA; 3 School of Applied Science, University of Science and Technology Beijing, Beijing, People’s Republic of China

**Keywords:** cell death, molecular target, organotins, proteasome, proteasome inhibitors, TPT

## Abstract

**Background:**

Because of the vital importance of the proteasome pathway, chemicals affecting proteasome activity could disrupt essential cellular processes. Although the toxicity of organotins to both invertebrates and vertebrates is well known, the essential cellular target of organotins has not been well identified. We hypothesize that the proteasome is a molecular target of environmental toxic organotins.

**Objectives:**

Our goal was to test the above hypothesis by investigating whether organotins could inhibit the activity of purified and cellular proteasomes and, if so, the involved molecular mechanisms and downstream events.

**Results:**

We found that some toxic organotins [e.g., triphenyltin (TPT)] can potently and preferentially inhibit the chymotrypsin-like activity of purified 20S proteasomes and human breast cancer cellular 26S proteasomes. Direct binding of tin atoms to cellular proteasomes is responsible for the observed irreversible inhibition. Inhibition of cellular proteasomes by TPT in several human cell lines results in the accumulation of ubiquitinated proteins and natural proteasome target proteins, accompanied by induction of cell death.

**Conclusions:**

The proteasome is one of the molecular targets of environmental toxic organotins in human cells, and proteasome inhibition by organotins contributes to their cellular toxicity.

In eukaryotes, more than 80% of intra-cellular proteins are degraded through the ubiquitin/proteasome-dependent pathway (Ciechanover 1998; Dou and Li 1999; Goldberg 1995). The ubiquitin/proteasome- dependent pathway plays an essential role in antigen presentation, cellular aging, apoptosis, and other major cellular processes. The cellular proteasome, commonly called 26S proteasome, is composed of two 19S regulatory particles and a 20S core particle. The latter is a multicatalytic threonine protease with at least three distinct catalytic activities: chymotrypsin (CT)-like (cleavage after hydrophobic residues mediated by the β5 subunit), trypsin-like (cleavage after basic residues by the β2 subunit), and peptidyl-glutamyl peptide-hydrolyzing (PGPH)-like (cleavage after acidic residues by the β1 subunit) (Goldberg 1995; Groll et al. 1997). Inhibition of proteasome CT-like activity by various compounds is associated with cell apoptosis (An et al. 1998; Lopes et al. 1997).

Since the 1960s, organotins, especially triphenyltin (TPT) and tributyltin (TBT), have been extensively used as antifouling boat paints, polyvinyl chloride stabilizers, agricultural pesticides, and industrial catalysts. Consequently, organotin contamination is found in various environmental media (Fent 1996). Because of their lipophilic property, organotins can be accumulated through the food chain and reach higher concentrations in top predators. For example, levels of organotins in stranded whales reached 1.0–1.1 mg/kg (Harino et al. 2007) and in the liver of harbor porpoises reached 68–4,605 mg/kg (Strand et al. 2005). Organotin chemicals have also been found in the tissues of humans contaminated by organotin insecticides or food (Kannan et al. 1999).

Exposure to TPT or TBT can also affect sex differentiation, resulting in masculinization of females or infertility in males (McAllister and Kime 2003; Smith 1996). TBT at low concentrations is toxic to cortical neurons by triggering glutamate excitotoxicity (Nakatsu et al. 2006). TBT also induces the differentiation of adipo-cytes *in vitro* and increases adipose mass *in vivo*, perhaps through activation of the retinoid X receptor and the peroxisome-proliferator– activated receptor γ (Grun et al. 2006). Both TPT and TBT can interfere with the cytotoxic function of natural killer (NK) cells (Snoeij et al. 1987), associated with increased cancer incidence. However, the toxicologic mechanism for organotin compounds is not completely understood, and the essential cellular target of organotins has not been identified.

The authentic proteasome inhibitor clasto-lactacystin βlactone contains an electrophilic-ester bond carbon that is responsible for its biological inhibition of the proteasome (Fenteany et al. 1995). We hypothesize that the electrophilic tin (Sn) atom in organotins could also be attacked by the O^γ^ atom of the N-terminal threonine (Thr-1) of the proteasome β5 subunit, causing irreversible inhibition. This hypothesis is supported by the present results from *in silico* docking, by *in vitro* proteasome activity assay using purified 20S proteasomes and human breast cancer MDA-MB-231 cells and human peripheral blood Jurkat T cells treated with TPT and other organotins, by analysis of CT-like activity, β5 proteasome subunit expression, and Sn levels in isolated proteasome complexes from the treated cells.

## Materials and Methods

### Materials

We obtained organotin compounds monophenyltin trichloride (MPT), diphenyltin dichloride (DPT), triphenyltin chloride (TPT), tetraphenyltin (TePT), monobutyltin trichloride (MBT), dibutyltin dichloride (DBT), tributyltin chloride (TBT), and tetrabutyltin (TeBT) from Acros Organics (Morris Plains, NJ, USA). A 50-mmol/L stock solution of each organotin [dissolved in methanol for all except TeBT and TePT; TeBT dissolved in dimethyl sulfoxide (DMSO), and TePT dissolved first in tetrahydrofuran and then diluted with an equal volume of DMSO], was stored at −20°C. Purified rabbit 20S proteasomes and fluorogenic peptide substrates Suc-LLVY-AMC, Z-GGL-AMC (for CT-like activity), Z-LLE-AMC (for PGPH-like activity), and Z-ARR-AMC (for trypsin-like activity) as well as Ac-DEVD-AMC (for caspase-3/7 activity) were from Calbiochem Inc. (San Diego, CA, USA). Rabbit antibody to 20S proteasome subunit β5, agarose-immobilized mouse monoclonal antibody to the 20S proteasome subunit α2 (HC3), and mouse antibody against human poly(ADP-ribose) polymerase (PARP) were purchased from BIOMOL International (Plymouth Meeting, PA, USA). Antibodies against Bax (B-9), p27 (F-8), ubiquitin (P4D1), actin (C-11), and IκB-α (C15) were from Santa Cruz Biotechnology, Inc. (Santa Cruz, CA, USA). The Apo-Direct Kit was from BD Biosciences Pharmingen (San Jose, CA, USA), and agarose-immobilized aprotinin and Ultrafree-MC centrifugal filter unit [M0286; pore size, 5-kDa molecular-weight (MW) cutoff] were from Sigma-Aldrich (St. Louis, MO, USA).

### Cell cultures, cell extract preparation, and Western blot analysis

We cultured human breast cancer MDA-MB-231 cells and human peripheral blood Jurkat T cells and prepared whole-cell extracts as previously described (An et al. 1998). Western blot analysis using the enhanced chemiluminescence reagent was performed as previously described (An et al. 1998).

### Proteasome activity assays using purified 20S proteasomes in intact cells

We measured the inhibition of purified 20S proteasomal activity (An et al. 1998) and 26S proteasomal activity in living intact cells (Chen et al. 2005) as described previously.

### Proteasome and caspase-3 activity assays using cell extracts

The prepared whole-cell extracts (10 μg per sample) from treated cells were incubated with the appropriate fluorogenic peptide substrates in 100 μL assay buffer at 37°C for 2 hr. We measured the release of the 7-amino-4-methylcoumarin (AMC) groups as previously described (An et al. 1998).

### Trypan blue dye exclusion assay and terminal deoxynucleotidyl transferase dUTP nick end-labeling (TUNEL) assay

We used the trypan blue dye exclusion assay to ascertain cell death in Jurkat T cells treated with TPT or TBT as described previously (Chen et al. 2005). The TUNEL assay using TPT-treated cells was performed with an APO-Direct kit to determine the extent of DNA strand breaks measured by flow cytometry according to the manufacturer’s instructions (An et al. 1998; Chen et al. 2005).

### Assay for interaction of TPT and purified 20S proteasome

We incubated purified rabbit 20S proteasomes (4 μg per reaction) with 10 μmol/L TPT or the control solvent methanol in 400 μL Tris-HCl buffer (25 mmol/L, pH 7.5) overnight at 4°C. The reaction mixture was then transferred to the insert cup of Ultrafree-MC centrifugal filter unit (MW cut-off, 5 kDa), which had been prewashed with 400 μL 1% bovine serum albumin,1% sucrose solution to minimize proteasome binding to the unit, and the mixture centrifuged at 7,000 rpm for 1.5 hr at 4°C, which resulted in about 80 μL of the proteasome preparation in the insert cup. Then 320 μL Tris-HCl buffer was added to the insert cup containing the proteasome preparation, followed by centrifugation again. This wash–filtration procedure was repeated four times to remove free TPT. The proteasome preparation in the insert cup was diluted to 100 μL with the Tris-HCl buffer and transferred to a storage tube. The insert cup was then washed with 50 μL Tris-HCl buffer and combined with the proteasome fraction. We used the prepared proteasome fraction for the proteasome activity and Western blot assays.

### Assay for interaction of TPT and cellular 26S proteasome

We treated MDA-MB-231 cells with 10 μmol/L TPT or control solvent methanol for 2 hr, followed by preparation of whole-cell extracts and the 26S proteasome immunoprecipitates. In brief, 100 μL agarose beads immobilized with mouse monoclonal antibody to the 20S proteasome subunit α2, or agarose beads immobilized with aprotinin (as a nonspecific binding control), were equilibrated with 1 mL buffer A (Tris-HCl buffer, 25 mmol/L, pH 7.5) and then incubated with 100 μL of the prepared cell extracts (~ 800 μg protein per preparation) overnight at 4°C in 700 μL buffer A. The reaction mixtures were centrifuged at 3,000 rpm for 1 min, the supernatants were removed, and the affinity matrixes were washed three times with 1 mL buffer A. The mixtures were resuspended with 400 μL buffer A and separated into two aliquots (200 μL each). One aliquot was diluted to 400 μL and incubated with Suc-LLVY-AMC (40 μmol/L) fluorogenic substrate at room temperature for 4 hr. After centrifugation at 3,000 rpm for 1 min, the supernatant was transferred to a 96-well plate for measurement of proteasome activity; the affinity matrix was resuspended with 100 μL 2× sodium dodecyl sulfate sample buffer, followed by Western blot analysis using proteasome β5 antibody. Another aliquot (200 μL) of the prepared proteasome immunoprecipitates was digested with an equal volume of 6 mol/L HCl for determination of the total Sn by inductively coupled plasma–mass spectroscopy (ICP-MS).

### ICP-MS analysis of Sn bound to proteasome

We determined total Sn levels in the prepared proteasome complexes with an Agilent 7500ce ICP-MS (Agilent Technologies, Santa Clara, CA, USA). The operating conditions were as follows: radiofrequency power, 1,240 W; Argon plasma gas (15 L/min), carrier gas (0.8 L/min), and makeup gas (0.27 L/min); sampling depth, 8.5 mmol/L; operation mode, shield torch; acquired mass, 118; points/mass, 3.

### In silico models for TPT binding to proteasome β5 subunit

Because 99% of TPT is present as the neutral hydroxyl-complex form (TPT-OH) in physiologic conditions (pH 7.5) (White and Tobin 2004), the chemical structure of TPT was selected as (C6H5)3SnOH for docking studies. The crystal structure of the proteasome **β**5 subunit, the docking parameters, docking methods, and output analysis methods were the same as described previously (Smith et al. 2004). In brief, we first refined the molecule by performing an optimized geometry calculation saved in Protein Data Bank (PDB) files using the conversion filters in CAChe software (v 6.1) (Fujitsu America Inc., Beaverton, OR, USA). The output PDB files were imported into AutoDock 3.0 software (The Scripps Research Institute 2008) for the *in silico* binding analysis to the proteasome β5 subunit. We chose the crystal structure of the proteasome β5 subunit of the eukary-otic yeast 20S proteasome (PDB reference no. 1JD2; RCSB Protein Data Bank 2008), which is similar to human 20S proteasome (Smith et al. 2004). We defined the Sn atom of TPT as a rigid atom and limited the docking space to a 20 × 20 × 20 Å box centered on the β5-catalytic N-terminal threonine that was prepared as an energy-scoring grid. The output from AutoDock was used for docking model studies with PyMOL software (Smith et al. 2004).

## Results

### Inhibition of purified 20S proteasomes by organotins

**Figure 1:**
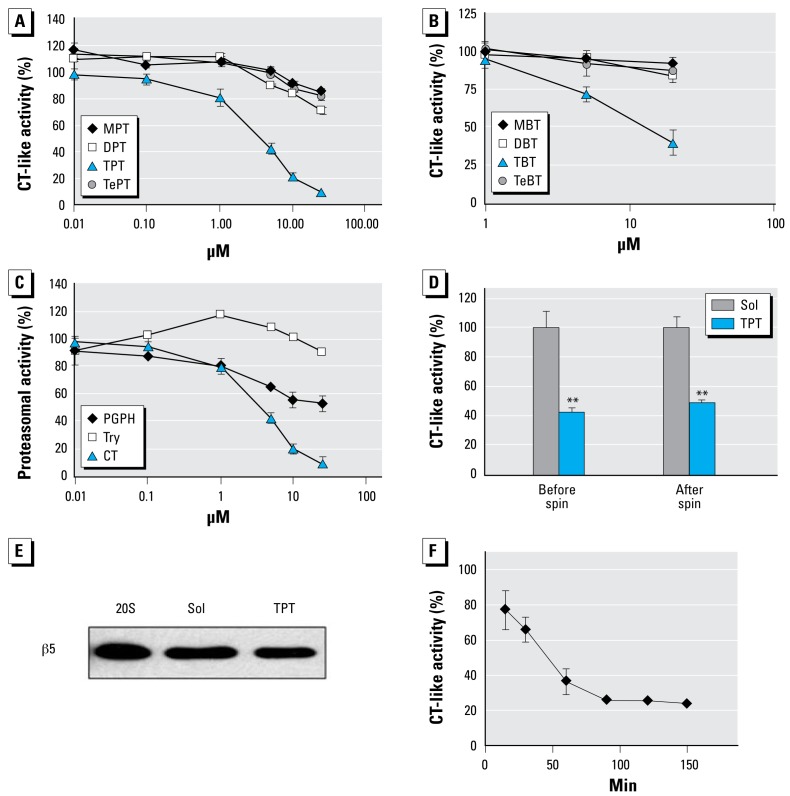
Inhibition of purified 20S proteasomes by organotins. Try, trypsin. Potency of phenyltins (*A*) and butyltins (*B*) to inhibit CT-like activity of purified 20S rabbit proteasomes. (*C*) TPT preferably inhibits CT-like activity of purified 20S rabbit proteasome. (*D* and *E*) Purified 20S proteasomes (4 μg) were incubated with 10 μmol/L of TPT or the solvent methanol (Sol). After removing the unbound TPT from the proteasomes in an Ultrafree-MC centrifugal filter unit (MW cutoff, 5 kDa), the proteasome preparations were measured for CT-like activity (*D*) and β5 subunit levels in Western blotting (*E*). (*F*) TPT at 10 μmol/L inhibited proteasomal CT-like activity in a time-dependent manner. Data are mean ± SD from three experiments. **p < 0.01.

We hypothesized that the proteasome is a major target of organotins. To test this hypothesis, we first investigated the inhibition potency of phenyltins and butyltins to purified 20S rabbit proteasomal activity. Among all the phenyltins tested (MPT, DPT, TPT, and TePT), TPT was the most potent inhibitor against proteasomal CT-like activity, with a half-maximal inhibitory concentration (IC_50_) of 3.5 μmol/L (Figure 1A). At 25 μmol/L, TPT and DPT caused 95% and 29% inhibition, respectively, whereas MPT and TePT had 15% or less inhibition (Figure 1A). When we tested butyltins with the purified 20S proteasome, we found a similar order of the CT-like-inhibitory activity: TBT (IC_50_ = 15.6 μmol/L) > DBT > MBT, TeBT (Figure 1B).

To investigate whether TPT specifically inhibits proteasomal CT-like activity, we examined its effects on the PGPH-like and trypsin-like activities of purified 20S proteasomes. At 10 μmol/L, TPT inhibited CT-like and PGPH-like activity of the purified 20S proteasomes by 79% and 44%, respectively, but had no inhibitory effect against trypsin-like activity (Figure 1C). Similarly, TBT had much less inhibitory effect on PGPH-like and trypsin-like activities of purified 20S proteasomes (data not shown). Therefore, it appears that organotins such as TPT and TBT preferentially inhibit proteasomal CT-like activity over other activities.

To further investigate the nature of TPT-mediated proteasome inhibition, we incubated the purified 20S proteasomes overnight at 4°C with either 10 μmol/L of TPT or control solvent. CT-like activity assays using an aliquot of each preparation indicated that TPT inhibited proteasome activity by 57% under these experimental conditions (Figure 1D, “before spin”). Most of the reaction mixtures were then washed several times with an Ultrafree-MC centrifugal filter unit (MW cutoff, 5 kDa) to remove small molecules, including free TPT. The proteasomal activity assay shows that repeated washing and filtration of the TPT-proteasome mixture did not change the outcome: TPT still caused 51% inhibition of proteasomal CT-like activity (Figure 1D, “after spin”). The lower level of the proteasomal activity detected in the TPT–20S proteasome mixture was not due to decreased levels of 20S proteasomes because an equal level of β5 subunit protein was detected by Western blotting in the two preparations (Figure 1E). This result indicates that TPT is either a tight-binding or an irreversible inhibitor of the proteasome.

### Kinetics of proteasome inhibition by TPT

Our data (Figure 1F) indicate that TPT inhibited proteasomal CT-like activity in a time-dependent manner: by 23%, 35%, 64%, and 76%, respectively, after 15, 30, 60, and 150 min. Because the observed proteasome- inhibition kinetics are characteristic for a mechanism-based inhibitor (Vicentini et al. 2001), we suggest that the TPT-mediated proteasome inhibition is irreversible.

### Computational study of TPT–proteasome β5 interaction

**Figure 2:**
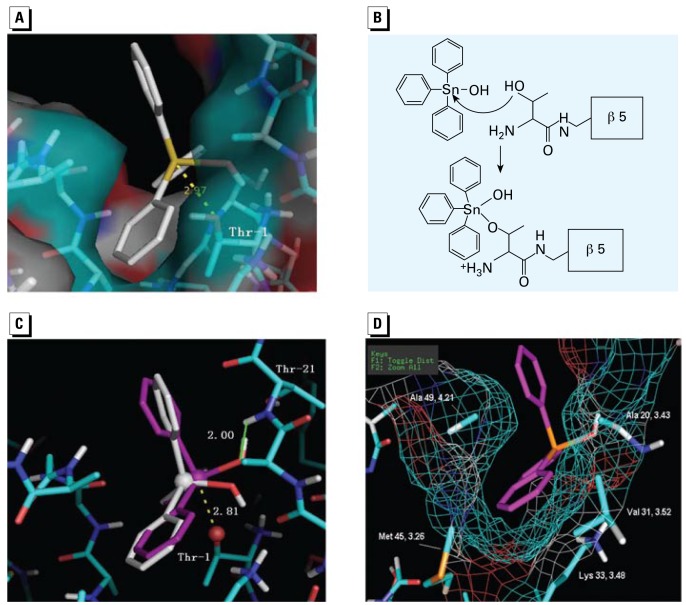
Docking studies for the interaction of TPT and the proteasome β5 subunit. (*A*) The docking model of TPT-OH with a tetrahedral conformation in the proteasomal β5 CT site; TPT-OH is represented by a stick structure. (*B*) A hypothetical mechanism for the interaction between TPT-OH and the β5 Thr-1 residue. (*C*) Comparison of TPT-OH with tetrahedral conformation (white) and trigonal bipyramidal conformation (pink) in the β5 site. The three phenyl rings, one OH legend, and O^γ^ on Thr-1 form the trigonal bi pyramidal conformation; the Sn on TPT and O^γ^ of Thr-1 are shown as spheres. (*D*) The hydrophobic interaction between the phenyl ring of TPT and the hydrophobic side chains in the S1 pocket. The mesh represents the surface of the S1 pocket.

In order to build a model to explain how TPT inhibits proteasomal CT-like activity, we performed automated docking studies. The docking results indicate that TPT has a major docking mode that repeated for 47 of 100 runs (47% probability). The distance from the Sn atom to the O^γ^ on Thr-1 was 2.97 Å, and one of the phenyl rings of TPT was located within the S_1_ hydrophobic pocket of β5 subunit (Figure 2A). It is well documented that the Thr-1 O^γ^ atom could be activated by Thr-1 N directly or via a neighboring water molecule, and reacts with the electrophilic groups of inhibitors (Groll et al. 1997). Because the Sn atom in TPT was close to the Thr-1 O^γ^ atom, we hypothesized that the Thr-1 O^γ^ atom might perform a nucleophilic attack on the Sn atom and form a coordinate bond (Figure 2B). It has been reported that with the attack of a nucleophilic ligand, the Cl ligand on the Sn atom of TBT or TPT could be replaced by the nucleophilic ligand, or the hybridization state of the Sn atom could change from sp3 to dzsp3, which allows the Sn atom to form a new coordinate bond with the nucleophilic ligand (Burda et al. 2002).

After the nucleophilic attack by Thr-1 O^γ^, the TPT– Thr-1 complex could be present in two possible conformations: tetrahedral (Sn in sp3 hybridization) and trigonal bipyramidal (Sn in dzsp3 hybridization). To determine whether the hybrid orbit orientation of Sn atom and the three-dimension space around Thr-1 O^γ^ could allow the formation of any of these conformations, we performed further docking studies. To mimic the coordinate complex of TPT–Thr-1, a threonine amide group was used as one of the ligands for Sn. The complex structure was refined by performing an optimized geometry calculation in MOPAC using PM5 parameters in the CAChe software. After optimization of the geometry, the threonine amide ligand was removed and the remaining structure was docked into the proteasome β5 subunit with AutoDock 3.0 software. The docked results indicate that TPT has one major trigonal bipyramidal conformation (Sn in a five-coordinate form; Figure 2C) that allows the formation of a coordinate bond. In contrast, the tetrahedral conformation (Sn in a four coordinate) does not give an orientation that allows the Sn and Thr-1 O^γ^ to form a coordinate bond (data not shown).

In the major trigonal bipyramidal conformation mode that repeated in 63 of 100 runs (63% probability), the OH ligand and two phenyl rings of TPT were on the equatorial orbit, whereas the third phenyl ring of TPT and the Thr-1 O^γ^ of β5 was on the axis orbit (Figure 2C). The distance between Sn and O^γ^ was 2.81 Å, and the line between Sn and O^γ^ was basically on the axis orbit orientation (Figure 2C), supporting that a coordinate bond could form between Sn and O^γ^. Also, one of the TPT hydrophobic phenyl ligands was oriented in the S_1_ pocket of β5 subunit (Figure 2D). The hydrophobic portion of the aromatic phenyl ring is oriented in the middle of the S_1_ pocket, with distances of 4.21 and 3.48 Å, respectively, to the side chains of Ala-49 and Lys-33 (Figure 2D). In addition, the sidewalls of the S_1_ pocket, which possibly interact with TPT hydrophobically, are created by Met-45, Ala-20, and Val-31 with distances of 3.26, 3.43, and 3.52 Å, respectively (Figure 2D). We also found that the distance between the amide hydrogen of Thr-21 and the oxygen on the hydroxide ligand of TPT is only 2.00 Å (Figure 2C), indicating the possible formation of a hydrogen bond. Therefore, the irreversible inhibition nature of TPT is supported by the docking results, the possible formation of a coordinate bond between Sn of TPT and Thr-1 O^γ^, blockage of the S_1_ pocket by a TPT phenyl ring, the possible hydrophobic interactions, and hydrogen bond formation.

### Organotins cause cellular proteasome inhibition and cell death

**Figure 3:**
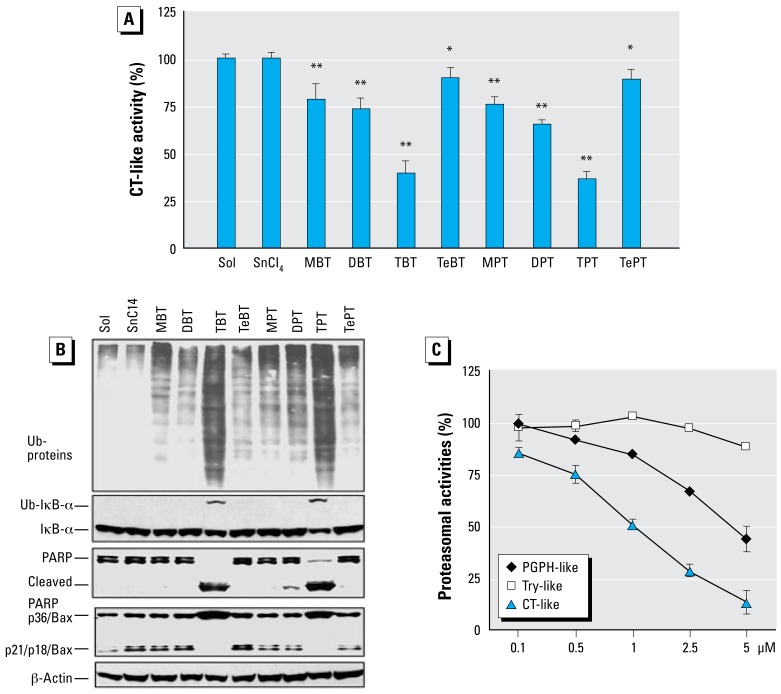
The proteasome-inhibitory and cell-death–inducing activities of organotins in MDA-MB-231 cells. Try, trypsin. (*A*) Proteasomal CT-like activity measured in intact MDA-MB-231 cells treated with the solvent methanol (Sol), inorganic Sn salt (SnCl_4_), or organotins (butyltins and phenyltins) at 10 μmol/L for 3 hr, followed by 1 hr additional incubation with 40 _mol/L of Z-CGL-AMC. (*B*) Western blot assay of ubiquitinated (Ub-)proteins, Ub-IκB-α, PARP, and Bax proteins in extracts from MDA-MB-231 cells treated with 10 μmol/L of organotins, SnCl_4_, or control for 4 hr. (*C*) Preferential inhibition of CT-like activity of cellular proteasome by TPT. Data are mean ± SD of three experiments. *p < 0.05. **p < 0.01.

To investigate whether the organotins could inhibit cellular proteasomal activity in intact cells, we chose human breast cancer MDA-MB-231 cells as a working model. The cells were plated in a 96-well plate and then treated with 10 μmol/L of each butyltin or phenyltin compound, SnCl_4_ (an inorganic tin as a comparison), or solvent (as a control) for 4 hr and the proteasomal CT-like activity was measured. We found that TPT was most potent among all the organotins tested and caused 63% proteasome inhibition under the experimental condition, whereas DPT, MPT, and TePT inhibited the proteasomal CT-like activity by 35%, 24%, and 11%, respectively (Figure 3A). Therefore, the rank of potency to inhibit cellular proteasome activity by organic phenyltins was: TPT > DPT > MPT > TePT, consistent with that of these phenyltins to inhibit the purified 20S proteasomal CT-like activity (compare Figures 3A and 1A).

Similarly, butyltins TBT, DBT, MBT, and TeBT inhibited the cellular proteasomal CT-like activity by 60%, 27%, 21%, and 10%, respectively (Figure 3A). The rank of potency in butyltins was TBT > DBT > MBT > TeBT, again similar to that of their inhibitory activities to the purified proteasomes (Figure 1B).

To verify the cellular proteasome-inhibitory ability of the organotins, MDA-MB-231 cells were grown on 100-mm dishes and then treated under the same condition (10 μmol/L of each butyltin or phenyltin compound for 4 hr). After the treatment, cells were collected and the cell extracts were prepared for analysis of proteasome inhibition by measuring accumulation of ubiquitinated proteins. We detected accumulation of ubiquitinated proteins mainly after treatment with TPT or TBT (Figure 3B). Other organotins had much less effect, whereas SnCl_4_ failed to increase the level of ubiquitinated proteins, compared with the solvent control (Figure 3B).

We have reported a ubiquitinated form of IκB-α protein with MW of about 56 kDa (Chen et al. 2007). A similar p56 band appeared after treatment of TPT or TBT, as detected by the specific antibody to IκB-α (Figure 3B, Ub-IκBα). In comparison, other organotins and SnCl_4_ showed no effect on accumulating the ubiquitinated IκBα protein (Figure 3B).

**Figure 4:**
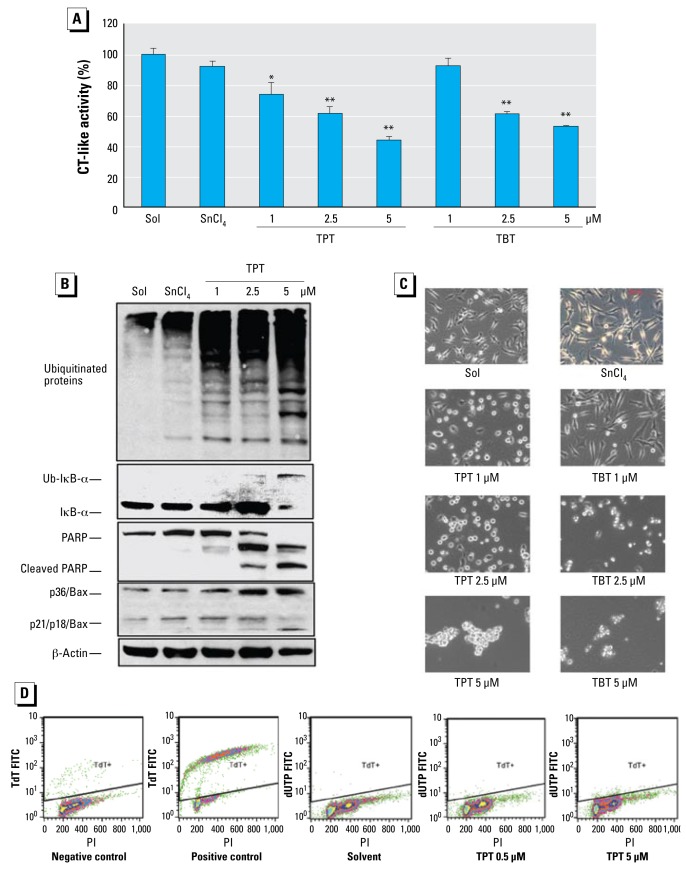
Dose effects of TPT and TBT on proteasome inhibition and cell death induction in MDA-MB-231 cells. Abbreviations: PI, propidium idodide; TdT FITC, terminal deoxynucleotidyl transferase fluorescein isothiocyanate. (*A*) Proteasomal CT-like activity measured in MDA-MB-231 cells exposed to either methanol (Sol), SnCl_4_ at 5 μmol/L, TPT, or TBT at the indicated concentrations for 6 hr (data are mean ± SD of three experiments). (*B*) Western blot assays of ubiquitinated proteins, ubiquitinated IκB-α (Ub-IκB-α), PARP, and Bax proteins levels in cell extracts. (*C*) Morphologic changes (100× magnifications; scale bar, 150 μm. (*D*) TUNEL assay. *p < 0.05. ** *p* < 0.01.

To investigate whether proteasomal inhibition by organotins is associated with cell death induction, we investigated both morphologic changes and PARP cleavage. Morphologic changes (shrinking, blebbing) were observed mainly in the MDA-MB-231 cells treated with TPT or TBT (data not shown, but see Figure 4C). Consistently, treatment with TPT or TBT caused the disappearance of the intact PARP protein (116 kDa), associated with production of a cleaved PARP fragment (Figure 3B). In contrast, other organotins were weaker cell death inducers and generated mild morphologic changes (data not shown). Only DPT treatment caused a low level of PARP cleavage; other organotins and inorganic SnCl_4_ had no effect (Figure 3B).

We and others have shown that, associated with apoptotic commitment, Bax protein (p21/Bax) could be cleaved by calpain, producing a p18/Bax fragment that then forms a homodimer p36/Bax (Gao and Dou 2000; Wood and Newcomb 2000). Treatment of MDA-MB-231 cells with TPT and TBT also caused an increase in levels of p36/Bax, associated with decreased levels of p21/p18/Bax (Figure 3B). In contrast, other organotins had much less effect (Figure 3B).

We then examined whether TPT and TBT could specifically inhibit the proteasomal CT-like activity in intact cells. MDA-MB-231 cells were treated with TPT or TBT at indicated concentrations for 12 hr, followed by measuring the three proteasomal activities in cell lysates prepared. At 2.5 μmol/L, TPT inhibited the CT-like, PGPH-like, and trypsin-like activities of the cellular proteasomes by 72%, 33%, and 3%, respectively (Figure 3C). Similarly, TBT also preferentially inhibited the CT-like activity over two other activities of the cellular proteasomes (data not shown). This result confirms that both TPT and TBT selectively inhibit proteasomal CT-like activity.

A 6-hr treatment of MDA-MB-231 cells with TPT at 1.0, 2.5, and 5.0 μmol/L also caused a dose-dependent inhibition (by 30%, 40%, and 58%, respectively) of the proteasomal CT-like activity (Figure 4A). Similarly, treatment for 6 hr with TBT at 1.0, 2.5, and 5.0 μmol/L inhibited 8%, 39%, and 46% of the cellular proteasomal CT-like activity, respectively (Figure 4A). Consistent with that, we detected increased levels of polyubiquitinated proteins, IκBα, and the ubiquitinated form of IκBα in a dose-dependent manner in the cells treated with TPT (Figure 4B) and TBT (data not shown), compared with the cells treated with inorganic SnCl_4_ or solvent. For example, levels of unmodified IκBα protein were increased by TPT at 1 and 2.5 μmol/L, and the ubiquitinated IκBα protein was accumulated by TPT at 2.5 μmol/L and was further increased by TPT at 5 μmol/L, associated with a decreased level of unmodified IκBα protein (Figure 4B). Furthermore, cell death also occurred in a dose-dependent fashion in the cells treated with TPT or TBT, as supported by cellular morphologic changes (Figure 4C) and PARP cleavage into p85 and p65 fragments (Figure 4B). We also observed cleavage of p21/Bax into p18/Bax and accumulation of p36/Bax in the cells treated with TPT (Figure 4B). These results demonstrate that TPT and TBT can inhibit cellular proteasomal CT-like activity, resulting in activation of cell-death–associated proteases. However, treatment with either TPT (Figure 4D) or TBT (data not shown) at all the tested concentrations did not cause DNA strand breaks, as evidenced by negativity of TUNEL assay, suggesting that organotin-induced cell death does not involve DNA damage.

### Kinetic studies of proteasome inhibition by TPT

**Figure 5:**
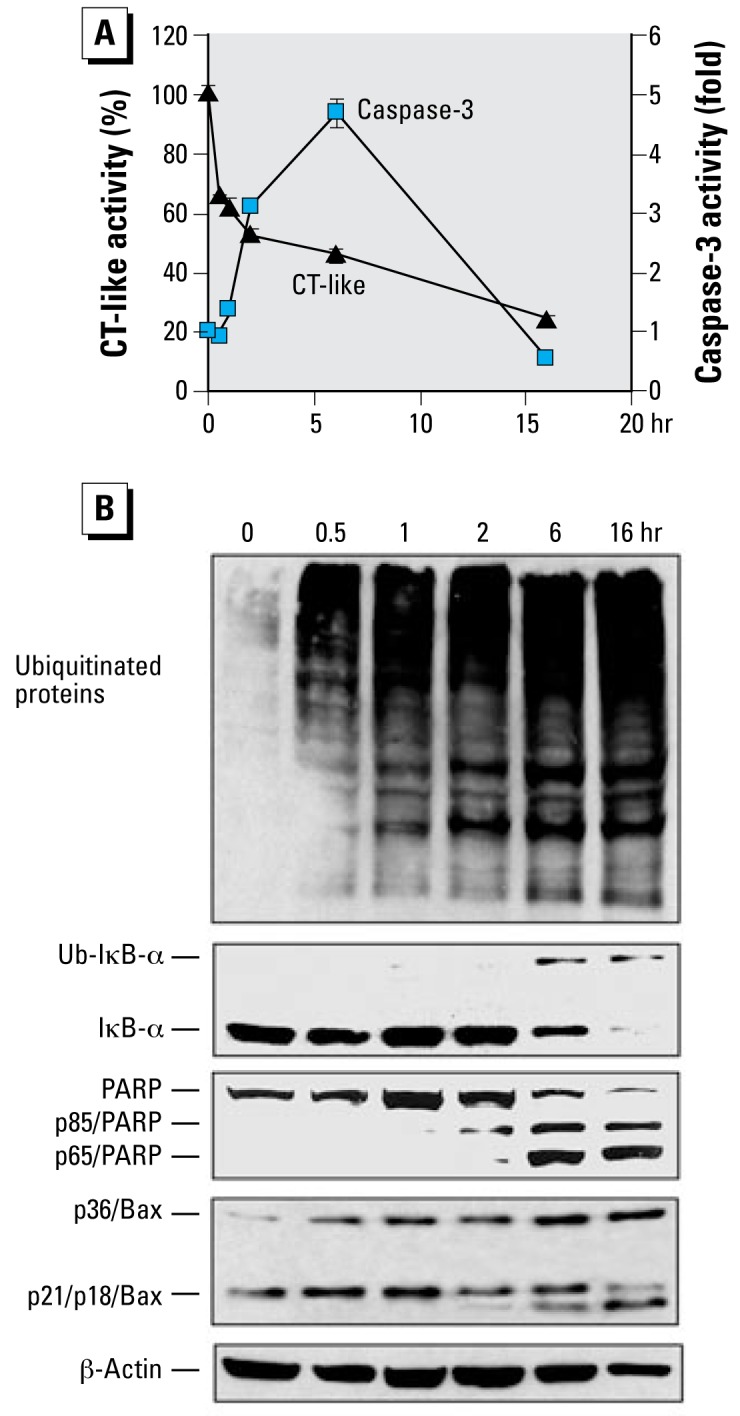
Kinetic studies on proteasome inhibition and cell death induction by TPT. (*A*) Exponentially grown MDA-MB-231 cells (0 hr) were exposed to 5 μmol/L of TPT for the indicated hr, and proteasome CT-like (triangles) and caspase-3 (squares) activities were measured. Data are mean ± SD of three experiments. (*B*) Western blot analysis of samples from the same experiment using specific antibodies to ubiquitin, IκB-α, PARP, Bax, and β-actin.

To determine whether proteasome inhibition or cell death is induced first by organotins, we treated MDA-MB-231 cells with 5 μmol/L of TPT for different time points, followed by measurement of proteasome inhibition and cell death. The proteasome inhibition by TPT started as early as 30 min, as shown by 35% inhibition of the proteasomal CT-like activity and accumulation of ubiquitinated proteins (Figure 5A,B). By 1 hr, proteasome activity decreased by 40% (Figure 5A) and levels of ubiquitinated proteins were further increased (Figure 5B). The ubiquitinated proteins accumulated until the last time point (16 hr, Figure 5B). Although levels of unmodified IκBα protein increased between 1 and 2 hr, that of ubiquitinated IκBα protein accumulated between 6 and 16 hr (Figure 5B). Importantly, cell death was not observed before 2 hr treatment with TPT, as shown by lack of caspase-3/7 activation (Figure 5A), PARP cleavage (Figure 5B), and cellular morphologic changes (data not shown). Only between 2 hr and 6 hr treatment with TPT was caspase-3/7 activity increased (by 3- to 4-fold; Figure 5A) and strong PARP cleavage bands detected (Figure 5B). Calpain activation was also found after 2 hr treatment, as shown by the increased levels of p65/PARP (Pink et al. 2000) and p18/Bax, as well as p36/Bax (Figure 5B). These results suggest that proteasome inhibition by TPT contributes to cell death induction.

### Organotins inhibit proteasome activity in multiple human cell lines

**Figure 6:**
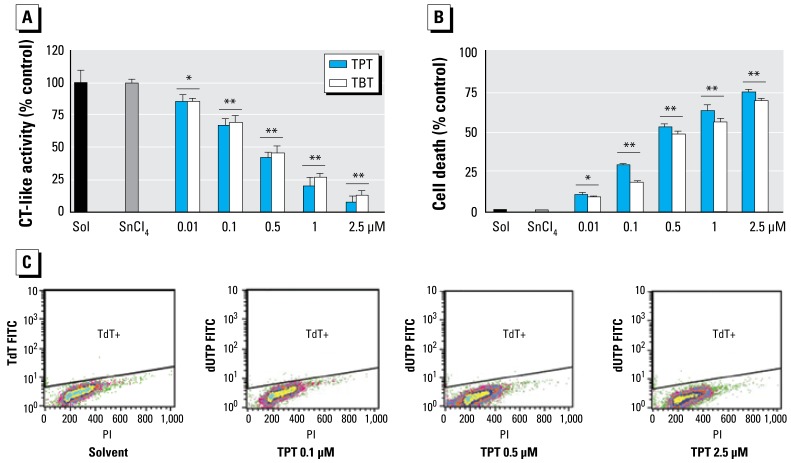
Dosage effects of TPT and TBT on proteasome inhibition and cell death in Jurkat T cells. Jurkat T cells were exposed to either methanol (Sol), SnCl_4_ at 5 μmol/L, or TPT and TBT at the indicated concentrations for 8 hr. (*A*) Measured proteasome CT-like activity. (*B*) Percentage of cell death measured by trypan blue assay. (*C*) TUNEL assay. Data are means ± SD of three experiments. *p < 0.05. **p < 0.01

One of the *in vivo* targets of organotins is the immune system. We therefore investigated whether organotins can inhibit proteasome activity in human peripheral blood Jurkat T cells. Jurkat cells were treated with TBT and TPT at concentrations ranging from 0.01 to 2.5 μmol/L for 8 hr, followed by measurement of the proteasomal CT-like activity, cell death, and TUNEL assays. TPT inhibited 14%, 33%, 58%, 79%, and 91% of proteasomal CT-like activity at concentrations of 0.01, 0.1, 0.5, 1, and 2.5 μmol/L, respectively, and TBT showed similar dose-dependent inhibition (Figure 6A). Again, SnCl_4_ at 5 μM had no inhibitory effect (Figure 6A). Proteasome inhibition in Jurkat cells treated with TPT and TBT was accompanied with dose-dependent cell death: TPT induced 12% and 77% cell death at 0.01 and 2.5 μmol/L, respectively, whereas TBT induced 10% and 71% cell death at 0.01 and 2.5 μmol/L (Figure 6B). Again, SnCl_4_ at 5 μM had no such effect (Figure 6A,B). TUNEL assay showed again that both TBT and TPT did not cause DNA strand breaks in Jurkat T cells (Figure 6C and data not shown), confirming that organotin-induced cell death is DNA–damage independent. Similar results were also found in human prostate cancer LNCaP cells and human normal, nontransformed YT natural killer cells (data not shown).

### Direct binding of TPT to the cellular proteasome

**Figure 7:**
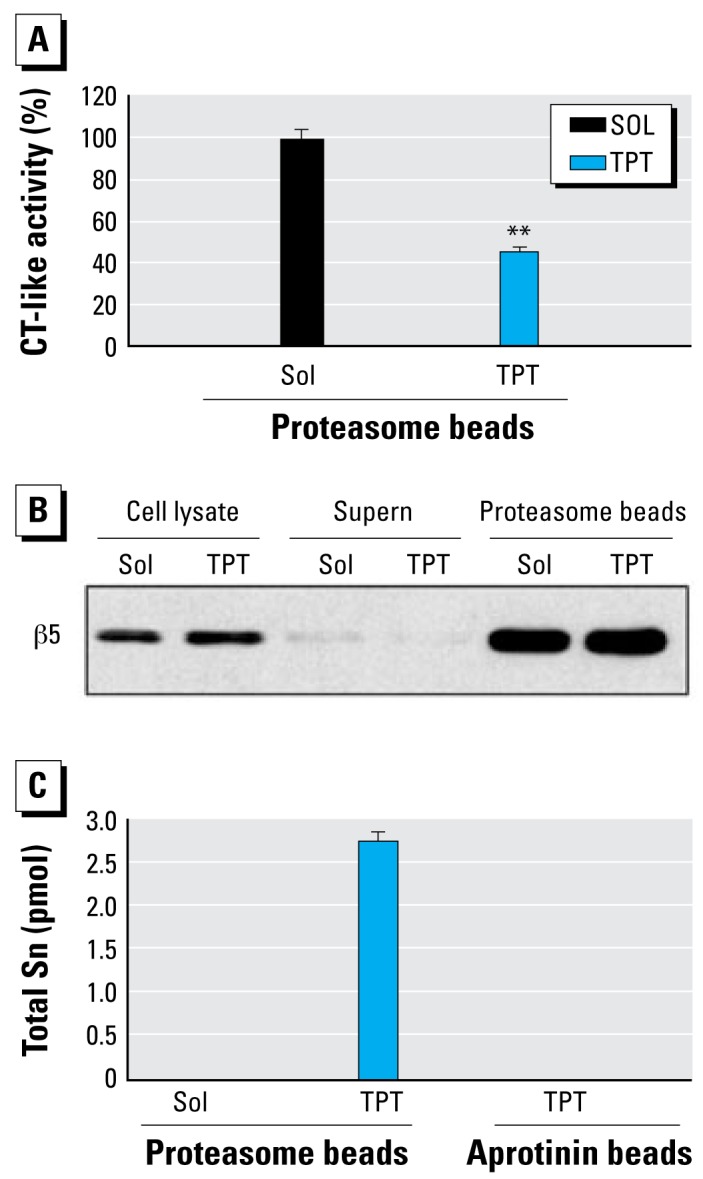
Direct interaction of TPT and the cellular proteasome. The 26S proteasome immunoprecipitates were prepared from MDA-MB-231 cells treated with 10 μmol/L TPT or methanol for 2 hr. The whole-cell extracts were immunoprecipitated with agarose beads conjugated with 20S proteasome subunit α2 antibody. Abbreviations: Sol, solvent; Supern, supernatant. (*A*) Associated proteasomal CT-like activity; (*B*) Proteasomal β5 protein level; (*C* ) Total Sn level measured by ICP-MS. Aprotinin immunocomplexes prepared from the cells treated with TPT were used as a nonspecific TPT binding control. Data are mean ±SD of three experiments. **p < 0.01.

To provide direct evidence for binding of TPT to cellular proteasome, we treated MDA-MB-231 cells with 10 μmol/L TPT or solvent for 2 hr, followed by preparing the proteasome immunoprecipitates using an antibody to the proteasomal α2 subunit. Aliquots of the prepared proteasome immunoprecipitates were used for measuring the associated proteasomal activity (Figure 7A), β5 subunit protein (Figure 7B), and the bound total Sn level (Figure 7C). A lower level of CT-like activity was found in the proteasome complexes prepared from the cells treated with TPT, compared with the control (Figure 7A), confirming the strong proteasome-inhibitory activity of TPT in cells. Equal protein levels of β5 subunit were found in the aliquots of the two proteasome immunoprecipitate preparations (Figure 7B), indicating that the decreased proteasome activity in TPT-treated sample preparation is not due to loss of the proteasome complex. Indeed, about 2.7 pM total Sn was found only in the aliquot of the proteasome complexes prepared from cells treated with TPT and not in the control (Figure 7C). As a nonspecific binding control, no Sn was detected in aprotinin complexes prepared from the above TPT-treated cells (Figure 7C).

Because TPT is relatively stable and can be slowly degraded to DPT, MPT, and inorganic tins only by human cytochrome P450 isoforms (Ohhira et al. 2006), and because only TPT at 10 μmol/L could cause 50% inhibition of proteasomal activity (Figures 3A, 4A), we believe that most cellular Sn detected in the proteasome immunoprecipitates under our experimental condition (within 2 hr treatment) should be TPT. Therefore, we conclude that the detected Sn represents TPT binding to the proteasome, which is responsible for the decreased proteasomal CT-like activity in the cells. These data strongly suggest that the proteasome is a direct cellular target of TPT.

## Discussion

In the present study, we have provided several lines of evidence that suggest the proteasome as an important cellular target of environmental toxic organotins. We have shown that TPT binds the proteasomal CT β5 site by *in silico* docking analysis (Figure 2) and that TPT potently and preferentially inhibits CT-like activity of purified 20S proteasomes (Figure 1) and cellular 26S proteasomes (Figures 3–6) in an irreversible manner. We have also shown direct binding of TPT to the proteasome in cells (Figure 7).

We found that phenyltins could inhibit both purified (Figure 1A) and cellular proteasomal CT-like activity (Figure 3A). The rank of inhibition potency was TPT > DPT > MPT, TePT. The various proteasome-inhibitory potencies of these phenyltins were roughly associated with their abilities to increase ubiquitinated proteins and the ubiquitinated form of IκB-α (Figure 3B). Similar conclusions can be drawn for the butyltins.

It has been well documented that the interaction and modification of the OH group on Thr-1 of proteasome β5 subunit are critical for the irreversible inhibition of CT-like activity (Fenteany et al. 1995). Results from our docking study suggest that Sn present in TPT might coordinately interact with the O^γ^ atom of Thr-1 of the proteasome β5 subunit (Figure 2). This hypothesis was supported by our experimental data that TPT irreversibly inhibited proteasomal CT-like activity (Figure 1). We therefore proposed a mechanism for the interaction between TPT and proteasome β5 subunit in which the Sn atom in TPT could be attacked nucleophilically by Thr-1 O^γ^, and the hybridization state of Sn could then change from sp3 to dzsp3, which allows the Sn to form a new coordinate bond with Thr-1 O^γ^ (Figure 2). During this process, the positions of phenyl groups might need only a minor change from their original tetrahedral conformation (Figure 2C).

The following arguments are consistent with the idea that TPT-mediated proteasome inhibition is functional and responsible for the observed cell death. First, when MDA-MB-231 cells were treated with TPT (or TBT), proteasome inhibition, calpain or caspase activation, and cell death induction were increased in both a dose- and time-dependent manner (Figures 3–5). Second, proteasome inhibition occurred before cell death (Figure 5), as demonstrated by multiple assays. Finally, similar data were obtained in human peripheral blood Jurkat T cells and other cell lines (Figure 6 and data not shown).

The most significant finding of the present study is the detection of Sn in the isolated proteasome complexes from the cells treated with TPT (Figure 7). We found no inter action of Sn and proteasomes in proteasome immunoprecipitates we prepared from the cells treated with the control solvent (Figure 7C) or in aprotinin (a serine protease inhibitor) complexes we prepared from the cells treated with TPT (Figure 7C), demonstrating a specific interaction. Based on the proteasome inhibition potency of phenyltins and the known pharmacokinetics of TPT metallization, we conclude that the detected Sn in the isolated proteasome complexes represents the bound TPT. These data strongly suggest that TPT directly binds to the cellular proteasomal β5 subunit and inhibits CT-like activity in cells.

We found that TPT (and TBT) preferably inhibited CT-like activity of purified 20S proteasomes (Figure 1C) and cellular proteasomes (Figure 3C), suggesting that β5 subunit is a specific target for organotins. This finding is significant because inhibition of the proteasomal β5 CT-like activity is associated with cell apoptosis (An et al. 1998; Lopes et al. 1997). Indeed, cell death occurred after TPT or TBT treatment. It appears that organotins induce at least some type of apoptosis because caspase-3 activation and p85 PARP cleavage fragment were observed (Figures 4, 5). However, cell death seems to not involve DNA damage because TUNEL assays showed negative results (Figures 4, 6). Because calpain is associated with necrosis and is activated by TPT and TBT, we also suspected occurrence of necrosis under our experimental conditions. Taken together, we conclude that organotins can induce necrosis and some caspase- dependent, DNA-damage–independent cell death. Finally, because organotins have similar effect on LNCaP cells (containing wild-type *p53* gene), MDA-MB-231 (mutant *p53*) cells, and Jurkat T cells (mutant *p53*), we suggest that organotins kill cells via a p53-independent pathway. This hypothesis is consistent with our previous report about proteasome inhibitors inducing tumor cell death in a p53-independent manner (An et al. 1998).

Our present findings may also provide an explanation for some previous observations. TPT and TBT have been reported to induce apoptosis in various cell systems (Mundy and Freudenrich 2006; Stridh et al. 1999), and some potential targets of organotins include NF-κB (Marinovich et al. 1996) and Bax (Zhu et al. 2007). Our results suggest that these previous observations might be downstream of proteasome inhibition because degradation of both IκB and Bax is regulated by the proteasome pathway (Dou and Li 1999; Li and Dou 2000). Furthermore, exposure to TPT or TBT has been shown to induce imposex, a pseudohermaphroditic condition, in female sea animals (Smith 1996). Because cytochrome P450 aromatase is the critical enzyme to catalyze the conversion of androgen to 17β-estradiol in eukaryote cells, inhibition of aromatase activity by TBT or TPT was often assumed to be responsible for the development of masculinized organs in female animals (Spooner et al. 1991). We suggest that inhibition of aromatase activity observed in organotin-exposed animals is due to inhibition of the proteasomes because proteasome inhibition causes up-regulation of the transcription factors USF1/2 (upstream stimulatory factors 1 and 2), which in turn suppress transcription of the *hCYP19*/aromatase gene (Jiang and Mendelson 2005).

In summary, the results from our present study strongly suggest that the proteasome is one of the molecular targets of environmental toxic organotins in human cells. The connection of organotin exposure to cellular proteasome inhibition provides a novel mechanism for environmental influences on human or animal health.
